# Evaluation of human thyroid hormone receptor-antagonist activity in 691 chemical compounds using a yeast two-hybrid assay with *Saccharomyces cerevisiae* Y190

**DOI:** 10.1016/j.dib.2022.108303

**Published:** 2022-05-21

**Authors:** Ryo Omagari, Mayuko Yagishita, Fujio Shiraishi, Ryo Kamata, Masanori Terasaki, Takuya Kubo, Daisuke Nakajima

**Affiliations:** 1 Health and Environmental Risk Division, National Institute for Environmental Studies, Japan; 2 Department of Life and Environmental Science, Prefectural University of Hiroshima, Japan; 3 Laboratory of Toxicology, School of Veterinary Medicine, Kitasato University, Japan; 4 Graduate School of Arts and Sciences, Iwate University, Japan; 5 Department of Material Chemistry, Graduate School of Engineering, Kyoto University, Japan; 6 Graduate School of Pharmaceutical Sciences, Chiba University, Japan

**Keywords:** yeast two-hybrid assay, human thyroid receptor, antagonist activity, EDSP21

## Abstract

The human thyroid receptor (hTR)-antagonist activities of 691 compounds were evaluated using a yeast two-hybrid assay with *Saccharomyces cerevisiae* Y190 introduced hTRα and coactivator. In parallel, those YTOX tests were conducted to evaluate whether those compounds affected either antagonism or toxicity. This is the first report that focuses on the hTR-antagonist activity of many chemical compounds suspected to be endocrine disruptor. In this study, 46 compounds exhibited antagonist activity at 50% of the maximum activity (IC × 50) within 11–9940 nM. In particular, 10,10-Oxybisphenoxarsine, triphenyltin fluoride, triphenyltin hydroxide, and chlorothalonil had strong hTR-antagonist activities. This knowledge gained from the present study will boost chemical regulation strategies for human and wildlife health.

## Specifications Table

**Table:** 

Subject	Cell Biology
Specific subject area	Screening of chemicals with human thyroid receptor (hTR)-antagonist activity by a yeast two-hybrid assay.
Type of data	Figure and Raw data
How the data were acquired	*Bioassay*: Yeast two-hybrid assay. *Instrument*: Luminescencer JNR AB2100; Atto Corp., Tokyo, Japan. (Luminescence was read on a 96-well plate luminometer)
Data format	Analyzed (Figure), raw (Excel)
Description of data collection	The hTR-antagonist activities of 691 chemicals were evaluated by a yeast two-hybrid assay using *Saccharomyces cerevisiae* Y190 introduced hTRα and coactivator. The stock solutions of the 691 compounds were prepared with dimethyl sulfoxide (DMSO) and stored at −30°C in darkness.
Data source location	• *Institution*: Health and Environmental Risk Division, National Institute for Environmental Studies (NIES) • *City/Town/Region*: Tsukuba city, Ibaraki prefecture • *Country*: Japan
Data accessibility	Raw data was deposited at Mendeley data. Repository name: The hTR-antagonist activities of 691 chemicals Data identification number: 10.17632/546fbt7g8m.1 Direct URL to data: https://data.mendeley.com/datasets/546fbt7g8m/1

## Value of the Data

• The hTR-antagonist activity of 691 compounds was evaluated by a yeast-two hybrid assay.
• It was determined that 46 compounds have hTR-antagonist activity.
• TR-antagonist activity in surface water in the environment has been often reported. However, appropriate treatment for removing the TR-antagonist activity has not been proposed due to the lack of nomination of chemicals with TR-antagonist activity.
• This data would be helpful to determine contributors to TR-antagonist activity in environmental water samples.

## 1 Data Description

**Fig. 1:**
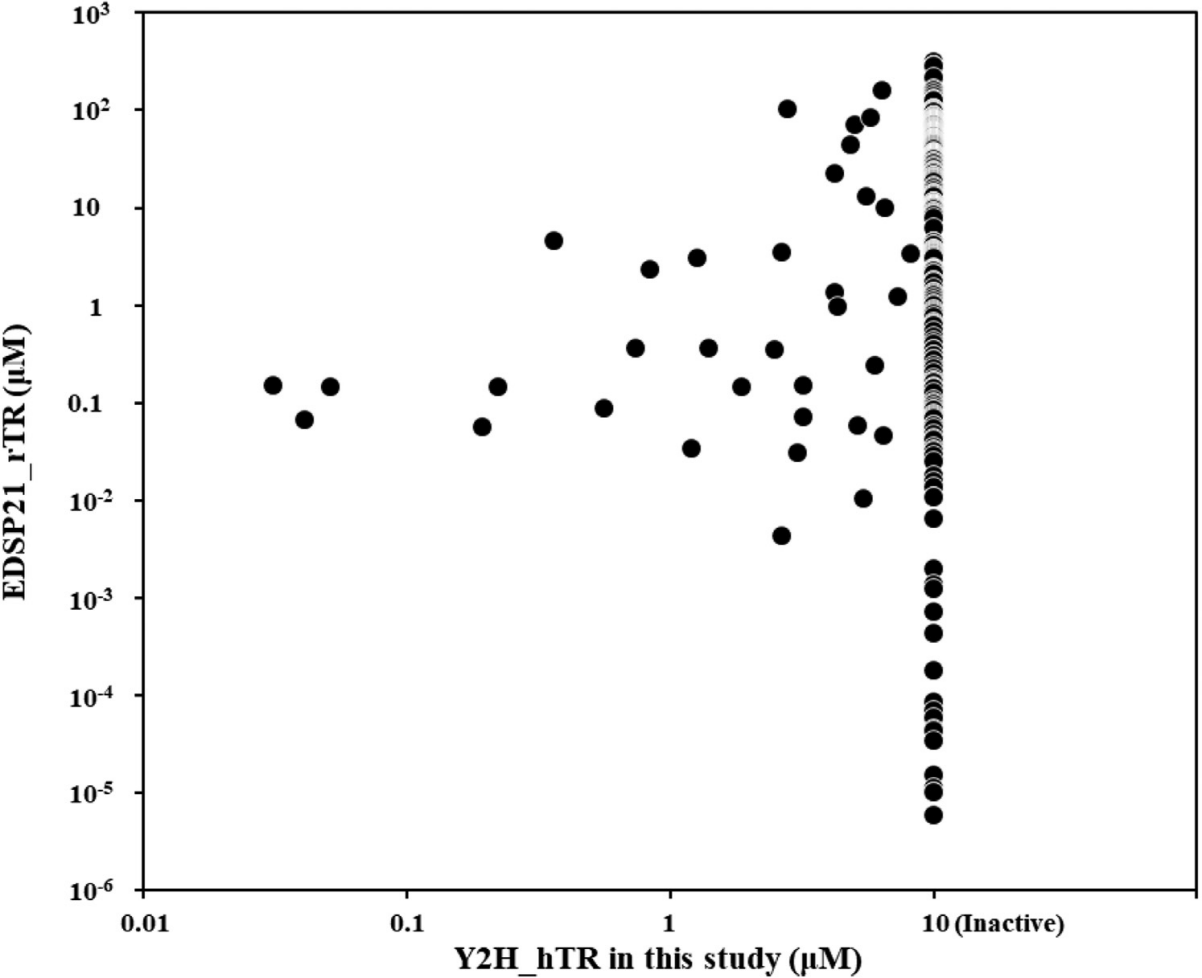
Comparison of the hTR-antagonist activity using the TR yeast-cell assay with rTR-antagonist activity using the EDSP21. If the hTR-antagonist activity was not defined in the test concentration range, the compound was considered as inactive for convenience. Those inactive values were described as “10 µM” in this study.

Fig. 1 presents a comparison of the hTR-antagonist activity by the TR yeast-cell assay with rat TR (rTR)-antagonist activity (AC_50_) by the Endocrine Disruptor Screening Program for the 21st Century (EDSP21) [1]. Of the 334 compounds investigated in both studies, none showed only hTR-antagonist activity, 298 compounds showed only the rTR -antagonist activity, and 36 compounds showed both hTR- and rTR-antagonist activities.

The 691 compounds with potential endocrine disrupting activity in raw data were selected as test substances, based on compounds with rTR-antagonist activity published in the EDSP21. In addition, we referred to the TR information in Extended Tasks on Endocrine Disruption 2016 (EXTEND 2016). Thus, most compounds suspected to have TR-antagonist activity were evaluated. 46 of the 691 compounds exhibited hTR-antagonist activity, while the remaining 645 compounds were evaluated as inactive substances for hTR. The raw data are available from the following link: https://data.mendeley.com/datasets/546fbt7g8m/1.

## 2 Experimental Design, Materials and Methods

Yeast cells (*Saccharomyces cerevisiae* Y190) with the human thyroid receptor (hTR)α and transcriptional intermediary factor-2 (TIF2) were utilized for evaluating an antagonistic binding activity of the 691 compounds to hTRα. In a yeast two-hybrid assay using the yeast cells (TR yeast-cell assay), the antagonist activity is defined by chemiluminescent intensity due to enzymic reaction with β-galactosidase; moreover, this assay is completed in a black 96-well culture plate [2], [3], [4]. A test procedure of the TR yeast-cell assay was performed as follows; 60 µL of modified SD medium including 40 nM of T3 with 0.2% DMSO (lacking tryptophan and leucine, 0.88% dextrose) was placed in all wells of the 96-well plate. A solution (the medium with 40 nM of T3 and 2% DMSO) including a test compound (60 µL) was added to the first-row wells, and then the test solution was serially diluted from rows 1 to 7 in duplicate. Next, 60 µL of suspension with the yeast cells, adjusted an absorbance of 1.75–1.84 at 595 nm after preincubation for 24 h at 30°C in the medium, was added to each well. Finally, the test concentration range was defined as 300–5,000 nM. In addition, the solvent control with 40 nM of T3 was also prepared in the eighth row of the 96-well plate. After adding the yeast suspension, the 96-well plates were incubated for 4 h at 30°C under high humidity, and then the living cell walls of the yeast cells were removed by lysis solution. The 96-well plates were put in an incubator at 37°C for the lysis reaction, and after 1 hour, chemiluminescence emitted from the free β-galactosidase by the substrate (Tropix Gal-Screen Substrate, Applied Biosystems) and enhancer (Sappire-II, Applied Biosystems) was measured using a 96-well plate luminometer (Luminescencer JNRAB2100, Atto Corp., Tokyo, Japan).

If a test compound exhibits inhibition of the chemiluminescence depending on the exposing concentrations, and the test compound doesn't have the toxicity to the yeast cells in a YTOX test; it was considered as a compound having the antagonist activity. The inhibition of the chemiluminescence was defined as a competition to the hTR-binding activity of 3,5,3′-triiodothyronine (T3), and the hTR-antagonist activity was calculated as a 50% inhibition concentration of the maximum activity (IC50). Metiram was employed as the positive control exhibiting the hTR-antagonist activity, and this result was utilized to conduct the quality control of the hTR-antagonism assay. If a test compound doesn't exhibit the chemiluminescence inhibition in the concentration range or has the toxicity to the yeast cells, it was considered as inactive compound or substance in the hTR-antagonist assay.

## Ethics Statements

This work does not include work involved with human subjects, animal experiments, and data collected from social media platforms

## CRediT Author Statement

**Ryo Omagari:** Validation, Formal analysis, Data curation, Writing – original draft, Visualization; **Mayuko Yagishita:** Validation, Writing – review & editing; **Fujio Shiraishi:** Methodology, Validation; **Ryo Kamata:** Validation, Writing – review & editing; **Masanori Terasaki:** Validation, Writing – review & editing; **Takuya Kubo:** Validation, Writing – review & editing; **Daisuke Nakajima:** Conceptualization, Validation, Writing – review & editing, Supervision, Project administration.

## Declaration of Competing Interest

The authors declare that they have no known competing financial interests or personal relationships that could have appeared to influence the work reported in this paper.

## Data Availability

The hTR-antagonist activities of 691 chemicals were evaluated by a yeast two-hybrid assay using Saccharomyces cerevisiae Y190 introduced hTRα and coactivator (Original data) (Mendeley Data).

## References

[1] The US Environmental Protection Agency (EPA). 2021. EDSP21 Dashboard. https://comptox.epa.gov/dashboard/chemical_lists/EDSPUOC.

[2] Shiraishi F., Kamata R., Terasaki M., Takigami H., Imaizumi Y., Yagishita M., Nakajima D. (2018). Screening data for the endocrine disrupting activities of 583 chemicals using the yeast two-hybrid assay. Data in Brief.

[3] Shiraishi F., Shiraishi H., Nishikawa J., Nishihara T., Morita M. (2000). Development of a simple operational estrogenicity assay system using the yeast two-hybrid system. [in Japanese]. Journal of Environmental Chemistry.

[4] Shiraishi F., Okumura T., Nomachi M., Serizawa S., Nishikawa J., Edmonds J.S., Shiraishi H., Morita M. (2003). Estrogenic and thyroid hormone activity of a series of hydroxy-polychlorinated biphenyls. Chemosphere.

